# Prevalence, Risk Factors, and Genetic Traits in Metabolically Healthy and Unhealthy Obese Individuals

**DOI:** 10.1155/2015/548734

**Published:** 2015-10-04

**Authors:** A. Berezina, O. Belyaeva, O. Berkovich, E. Baranova, T. Karonova, E. Bazhenova, D. Brovin, E. Grineva, E. Shlyakhto

**Affiliations:** ^1^Federal North-West Medical Research Centre, 2 Akkuratova Street, Saint Petersburg 194341, Russia; ^2^First Pavlov State Medical University, 6-8 L Tolstoy Street, Saint Petersburg 197022, Russia

## Abstract

*Objective*. To assess prevalence of metabolically healthy individuals among patients with abdominal obesity (AO) and to determine phenotype and potential genetic traits associated with a benign metabolic status. *Methods*. 503 AO patients without cardiovascular diseases were examined. Waist circumference (WC), BMI, blood pressure, plasma glucose and serum insulin levels, HOMA-IR, lipid profile, and adiponectin (AN) and leptin (LEP) concentrations in serum were measured. Polymorphisms A19G and Q223R of the LEP and LEP receptor gene, and G276T and T45G of the AN gene were investigated. *Results*. 91.3% of patients were metabolically unhealthy obese (MUO), and 8.7% metabolically healthy obese (MHO). MHO patients were younger, and had lesser BMI and WC, while duration of obesity, frequency, and duration of physical training were greater than MUO patients (*p* < 0.05). In MHO and MUO patients distribution of the G19G, G19A, and A19A genotypes of the LEP gene and G276G, G276T, and T276T genotypes of AN gene did not differ. The Т45Т genotype was associated with increase of metabolic disorders' risk for patients with АО (OR = 2.331; 95%  CI = 1.121 ÷ 5.132). *Conclusions*. Prevalence of MHO individuals among patients with AO is low. Benign metabolic status was associated with younger age, lower waist circumference, and higher physical activity, shorter duration of obesity, and G45G adiponectin genotype carriage.

## 1. Introduction

Abdominal obesity (AO) is one of the major risk factors for cardiovascular disease (CVD) and its complications [1]. On the one hand, this is thought to be the result of increasing prevalence of obesity in all age groups all over the world while, on the other hand, there is a distinct correlation between AO and early CVD and its complications, especially in patients with metabolic syndrome (MetS) [2, 3]. At the same time in a number of population studies, it has been shown that, despite AO, some subjects retained their insulin sensitivity and had normal lipid and glucose levels and blood pressure and cytokine profile and, accordingly, their risk of developing type 2 diabetes mellitus (T2DM) and CVD was rather low [4–7]. Such a subpopulation of obese individuals is usually called “metabolically healthy obese” (MHO). Data of different authors showed that the prevalence of this phenomenon in the population of obese individuals varies widely from 6.0% to 38.4%. Such inconsistencies are stipulated, first and foremost, by the absence of unified assessment criteria for this cohort. We could see that the “metabolic health” criteria in various studies were as follows: insulin sensitivity of the tissues measured by different methods and techniques, absence of the metabolic syndrome criteria based on diverse classifications, complete absence of any metabolic disorders, and, finally, at least 2 CVD risk factors [5–13].

Till now, the question of whether subjects with MHO have some specific phenotypic and/or genetic traits or they are at the intermediate stage of complicated obesity is still to be answered. A number of scientific papers showed that adipocytokine imbalance and genetic factors accounting for the expression of adipocytokines and/or polymorphism of the adiponectin gene as well as that of the leptin and its receptor gene might play a certain role in triggering or, vice versa, preventing metabolic disorders. That is why in our study we tried to identify possible factors including genetic ones that determine these individuals' benign metabolic profile.

*Objective*. The objective of this study is to assess the prevalence of metabolically healthy individuals among the subgroup of patients with abdominal obesity and to determine the phenotype and potential genetic traits associated with a benign metabolic status.

## 2. Methods

A total of 503 residents of Saint Petersburg with AO aged 30 to 55 (average age 45.8 ± 0.3 years) have been enrolled in the study. The inclusion criteria were abdominal obesity (AO), that is, waist circumference (WC) ≥94.0 cm and ≥80.0 cm for men and women, respectively, and signed informed consent (IDF criteria of MetS, 2005). Patients with CVD were not included in the study. Among screened participants, there were 359 (71.4%) women and 144 (28.6%) men of comparable age (45.1 ± 0.6 and 46.1 ± 0.4, resp.; *p* > 0.05). Mean WC was 98.41 ± 0.62 cm in women and 108.39 ± 0.86 cm in men.

In the course of this study, the following anthropometric parameters were measured in accordance with the conventional rules: height, weight, WC, waist-to-hip ratio (WHR), and body mass index (BMI). The subjects were interviewed about their sociodemographic status (education level, income, and family status), familial predisposition to obesity, cardiovascular disease and diabetes, smoking and alcohol consumption, birth weight, duration of obesity, and physical activity (frequency and duration of training sessions per week).

Fasting plasma glucose (FPG) was measured with the automated biochemistry analyzer (COBAS INTEGRA 400/700/800) and standard kits (Roche, Germany). High-sensitivity C-reactive protein (hsCRP) quantitative determination was performed by immunoturbidimetric assay. Serum lipids profile (total cholesterol (TC), triglycerides (TG), high-density lipoprotein cholesterol (HDL-C), and low-density lipoprotein cholesterol (LDL-C)) was measured by enzymatic colorimetric assays with the analyzer COBAS INTEGRA 400/700/800 (Germany) and standard kits (Roche, Germany). Serum insulin was measured by solid-phase enzyme immunoassay (ELISA) with the DRG standard test kits (EIA-2935) (USA). Insulin resistance was assessed by calculating insulin resistance index, homeostasis model assessment (HOMA-IR) [14]. Leptin and adiponectin concentrations in venous blood serum were measured by ELISA with the testing kits manufactured by “Leptin ELISA” (DRG Diagnostics, Germany) and the “DRG Adiponectin (human) ELISA” kits (DRG Diagnostics, Germany).

Genomic DNA was extracted from the peripheral blood lymphocytes by the modified method of Blin and Stafford (1976) [15]. Polymerase chain reaction (PCR) was performed with the automated MJ Research (MJ Research Inc.) and Biometra (Biometra, Germany) cyclers with thermostable recombinant Taq polymerase manufactured by Sibenzyme (Russia). Identification of leptin gene A19G polymorphism was done by PCR followed by restriction analysis [16]. For identifying leptin receptor gene (LEPR) Q223R polymorphism PCR followed by restriction analysis (Gotoda et al., 1997) was performed [17]. For identifying adiponectin gene G276T polymorphism the method by Křížová et al. (2008) was applied [18]. Adiponectin gene T45G polymorphism was identified by PCR followed by restriction analysis [19].

Studying polymorphisms of these genes was prompted by the availability of data suggesting their possible involvement in both obesity and glucose and lipid metabolism disorders, which allows us to consider them candidate genes potentially contributing to physiological and/or pathological processes.

“Metabolically healthy obese” (MHO) were defined as individuals without metabolic syndrome (MetS) according to IDF criteria of MetS and without insulin resistance according to HOMA-IR, while patients with MetS or other metabolic disorders were identified as “metabolically unhealthy obese” (MUO).

### 2.1. Statistical Analysis

Biomedical research data was processed by SPSS software 17.0 for Windows. Sample characteristics were presented as an average ± mean error. Frequency characteristics of qualitative variables were analyzed by nonparametric techniques with the use of *x* ^2^, Fisher criterion. Odds ratio (OR) was calculated to define relative risk of the disease. OR = 1 was considered as indicating no association, OR > 1 indicated positive association, and OR < 1 indicated negative association of an allele or genotype with the disease (lower disease risk). Confidence intervals for frequency characteristics were estimated by precise Fisher's method, *x* ^2^ with Yates' correction (for small groups). Comparison of quantitative parameters in the study arms was done by ANOVA. Studied parameters were compared for various classification methods and over-time evaluation techniques (paired samples) by applying the sign test, Wilcoxon signed-rank test, and Friedman test.

## 3. Results

Among 503 subjects with AO, BMI ≥ 30.0 kg/m^2^ was diagnosed in 57.0%. Subjects were divided into the following groups based on their BMI: 28 (5.6%) subjects had BMI ≤ 24.9 kg/m^2^; 180 (35.9%) patients had BMI ≤ 29. 9 kg/m^2^; 192 (38.2%) had BMI ≤ 34.9 kg/^2^; 70 (13.9%) had BMI ≤ 39.9 kg/m^2^; and 32 (6.4%) had BMI ≥ 40.0 kg/m^2^. Various MetS components were found in 91.3% of the patients with AO, 66.5% out of those who were diagnosed with MetS. Only 8.7% of the patients with AO did not have insulin resistance or MetS components. Thus among patients with AO whose waist circumference met the IDF criteria of MetS diagnosis, prevalence of metabolically healthy subjects turned out to be rather low.

Comparative analysis between MHO and MUO subjects has shown that MHO individuals were younger and had lower BMI, WC, WHR, levels of total cholesterol, LDL-C, triglycerides, insulin, HOMA-IR, glucose, C-reactive protein, blood pressure, and intima-media thickness of the common carotid arteries but higher HDL-C as opposed to MUO patients (Table 1). Average leptin and adiponectin levels did not differ between these groups (*p* > 0.05). However, in MHO women, adiponectin level was higher whereas that of leptin was lower than in MUO female.

We also determined that more than half of MHO individuals (58.1%) had had obesity for less than 6 years as opposed to 33.0% for MUO subjects (*p* = 0.002). Thus, MHO individuals had a shorter duration of obesity than MUO patients.

47.0% of MHO individuals and 33.4% MUO subjects practiced physical activity or had leisure-time exercise sessions (*p* > 0.05). In addition to this, the frequency of physical exercise sessions and their duration were significantly higher in MHO than in MUO patients (1.28 ± 0.31 times/week and 0.92 ± 0.11 times/week, resp.; *p* = 0.032; 27.70 ± 6.23 min/week and 15.81 ± 1.42 min/week, resp.; *p* = 0.040). This means that MHO individuals were more physically active than MUO.

There were no significant differences between the study arms in terms of social status, birth weight, number of meals, smoking and alcohol consumption status, familial predisposition to obesity, cardiovascular disease, and diabetes mellitus (*p* > 0.05).

Correlations between waist circumference, metabolic parameters, blood pressure, and adipocytokines were identified (Table 2).

Linear regression analysis has demonstrated that WC had made an impact on both systolic and diastolic blood pressure (*r* ^2^ = 0.212, *p* = 0.0001 and *r* ^2^ = 0.101, *p* = 0.0001, resp.), insulin (*r* ^2^ = 0.233, *p* = 0.0001), HDL-C (*r* ^2^ = 0.116, *p* = 0.0001), and leptin (*r* ^2^ = 0.338, *p* = 0.0001). Age affects BP (*r* ^2^ = 0.107, *p* = 0.0001), insulin (*r* ^2^ = 0.221, *p* = 0.0001), leptin (*r* ^2^ = 0.312, *p* = 0.0001), and adiponectin (*r* ^2^ = 0.119, *p* = 0.0001). Waist-to-hip ratio determines HDL-C (*r* ^2^ = 0.120, *p* = 0.0001) and adiponectin (*r* ^2^ = 0.117, *p* = 0.0001). Duration of physical activity influences blood pressure (*r* ^2^ = 0.201, *p* = 0.0001) and insulin (*r* ^2^ = 0.224, *p* = 0.0001).

At the same time, regression analysis has demonstrated that WC depended on sex, physical activity, duration of obesity, and number of meals.

Negative correlations were observed between adiponectin and TG (*r* = −0.234, *p* = 0.003), HOMA-IR (*r* = −0.212, *p* = 0.006), insulin (*r* = −0.231, *p* = 0.0001), WC (*r* = −0.214, *p* = 0.003), WHR (*r* = −0.203, *p* = 0.002), and BMI (*r* = −0.128, *p* = 0.030) whereas positive correlations were between adiponectin and HDL-C (*r* = 0.209, *p* = 0.003). In the course of regression analysis, it has been shown that the following indicators impacted adiponectin levels to a greater extent: insulin, WHR, and TG (*r* ^2^ = 0.115, *p* = 0.0001 for all parameters).

Conversely, positive correlations were detected between leptin and HOMA-IR (*r* = 0.108, *p* = 0.011), insulin (*r* = 0.211, *p* = 0.0001), WC (*r* = 0.326, *p* = 0.0001), and BMI (*r* = 0.405, *p* = 0.031). Regression analysis has shown dependence of the leptin level on BMI and TG (*r* ^2^ = 0.211, *p* = 0.0001).

Within the framework of this study, genotype distribution and alleles frequency of the leptin (019G polymorphism), leptin receptor (LEPR) (Q223R polymorphism), and adiponectin receptor (T45G and G276T polymorphisms) were analyzed in MUO and MHO as these genes potentially might be responsible for the development of obesity and metabolic disorders.

Distribution of genotypes and allele frequency of the leptin gene were analyzed in 459 metabolically healthy and unhealthy individuals with AO. In patients with and without metabolic disorders, distribution of the G19G, G19A, and A19A genotypes and frequency of the 19A and 19G alleles of the leptin gene did not differ (*p* > 0.05) (Table 3). Also, no differences between the groups of AO patients with and without metabolic disorders have been shown in analysis of the Q223Q, R223Q, and R223R genotypes and frequency of the 223Q and 223R LEPR alleles (Table 3).

Distribution of the G276G, G276T, and T276T genotypes and frequency of the 276T and 276G alleles of the adiponectin gene did not differ between MHO and MUO patients (Table 4).

In addition, there were more carriers of the 45T allele of the adiponectin gene among metabolically unhealthy patients with AO as opposed to metabolically healthy ones (Table 4). The T45T genotype was associated with over twofold increase of metabolic disorders' risk for patients with AO (OR = 2.331; 95% CI = 1.121 ÷ 5.132).

To make sure that both groups were comparable, we adjusted them to leave only overweight and class I obese patients based on the WHO classification, hence excluding all subjects with BMI ≥ 35.0 kg/m^2^ regardless of the gender, a process that leads to removing 102 MetS subjects from the analysis groups. This approach was adopted since there were no class II and higher obese patients in MHO group. The comparative results confirmed that there were more carriers of the 45T allele of the adiponectin gene among MUO as opposed to MHO subjects (frequency 45T allele: 0.934 and 0.864, resp.; *p* = 0.018). Hence T45T genotype carriage was associated with an increased risk of metabolic disorders in subjects with AO (OR = 2.496; 95% CI = 1.127 ÷ 5.524).

Average values of leptin and adiponectin in carriers of various genotypes of the leptin and leptin receptor genes were not different. In MHO and MUO men and women with various genotypes of the studied genes, levels of leptin and adiponectin were the same. However, in women with AO with and without MetS/metabolic disorders (general cohort), carriers of the 223R allele of the LEPR gene, leptin levels were higher than those in individuals with the Q223Q genotype (57.124 ± 0.370 ng/mL and 46.301 ± 2.752 ng/mL, resp.; *p* = 0.030). Furthermore, in women with AO and metabolic disorders with the R223R genotype, leptin levels were higher than those in women, carriers of the 223Q allele of the LEPR gene (60.744 ± 5.581 ng/mL and 47.521 ± 3.243 ng/mL, resp.; *p* = 0.045). Leptin levels did not differ (*p* > 0.05) in the cohort of MHO women with various genotypes of the leptin receptor gene.

## 4. Discussion

According to the results of the study, prevalence of such a phenomenon as absence of the metabolic syndrome and/or metabolic disorders in persons with AO, residents of Saint Petersburg of employable age, turned out to be insignificant and amounted to 8.7%. These individuals did not have any signs of the metabolic syndrome except for their waist circumference value in accordance with the IDF criteria and insulin resistance according to HOMA-IR. For comparison, study conducted by Iacobellis et al. (2005) has proven that in an Italian cohort of obese patients, in which the MetS IDF criteria were also used as a discriminator of complicated and uncomplicated obesity in combination with assessment of such parameters as uric acid, fibrinogen, and insulin levels, prevalence of this phenomenon was higher and amounted to 27.5% despite the fact that selection criteria were stricter than those in our study [12]. These differences might be due to a number of factors like specific features of the study subjects (number of evaluated patients, their ethnicity, age, obesity duration, etc.), criteria used, and, perhaps, genetic peculiar traits, nutrition patterns, physical activity, and other environmental factors.

In our study, characteristic phenotypic features of obese individuals without MS or metabolic disorders were defined. These include younger age, less pronounced abdominal obesity with lesser duration, and a higher level of physical activity as opposed to persons with AO and MetS/metabolic disorders. According to the obesity classification by BMI, all metabolically healthy individuals fell into the category of overweight patients even though their waist circumference met the IDF criteria of abdominal obesity. Currently, it is proven that both waist circumference and waist-to-hip ratio strongly correlate with the amount of the visceral fat. It is also known that the visceral adipose tissue generates large quantities of adipocytokines and anti-inflammatory substances that potentiate the development of insulin resistance and other metabolic disorders resulting in earlier debut of cardiovascular disease in obese patients. Presence of correlations between WC and metabolic parameters revealed in this study as well as other numerous studies confirms the relationship between AO and metabolic disorders. The obtained data is consistent with the results of other studies that also showed that individuals with uncomplicated obesity had less visceral fat than patients with obesity and metabolic disorders [20]. Brochu et al. have found that despite similar amount of total fat mass in postmenopausal obese women, MHO women had 49% less visceral adipose tissue than women with obesity and metabolic disorders.

Thus, lesser amount of the visceral adipose tissue may, to some extent, determine the favorable metabolic profile of this patient population [6].

In the vast majority of subjects with uncomplicated obesity that we evaluated, obesity duration was relatively short, less than 6 years. According to other studies' results, subjects suffering from obesity since childhood were encountered more frequently among MHO individuals. It is considered that children and adolescents develop hyperplastic obesity which is associated with preserved insulin sensitivity and with maintained adiponectin concentration, a combination determining the favorable profile of “metabolically” healthy obese individuals [6]. Another hypothesis was proposed by Muscelli et al. (1998), according to which early obesity was accompanied by some kind of metabolic adaptation enabling preservation of normal insulin sensitivity of the tissues [21]. It is also known that complicated obesity is often associated with ectopic fat depositions in the heart, liver, and muscles, which causes the development of insulin resistance and dysfunction of these organs. Conversely, uncomplicated obesity is characterized by a low degree of ectopic fat depositions especially in the muscles and liver [22].

Physical activity (PA) is a potential factor influencing the metabolic profile of obese patients. Thus, in our previous study, it was shown that in physically active AO patients with MS/metabolic disorders both insulin levels and insulin resistance index HOMA-IR were lower whereas the HDL-cholesterol level was higher compared with AO individuals with sedentary life styles while anthropometric indicators in these cohorts were comparable [23]. Therefore, PA can exert an independent positive influence on the metabolic profile of AO patients. Taking this into consideration, one may assume that MHO intense physical activity facilitates maintenance of the normal metabolic status, which is consistent with the results of the study carried out by Wildman et al. (2008) [9]. At the same time, other studies did not find any differences between the basal metabolic rate and energy expenditure on physical activity and level of physical performance in obese patients with the different metabolic profile [6, 22].

It is known that adipocytokine imbalance, in particular that of leptin and adiponectin, is associated with MetS. In a number of studies, it has been shown that an elevated leptin level potentiated insulin resistance and arterial hypertension and activated proinflammatory factors while adiponectin, on the contrary, possessed cardioprotective effects. This association is also supported by the results of correlation and regression analysis done in our study. However these adipokine levels are sex-dependent. For example, estimated levels of leptin and adiponectin in our study were lower in men than in women in both evaluated cohorts. Only metabolically healthy obese women had a lower leptin and higher adiponectin level as opposed to women with complicated obesity. That being said, it may be assumed that less significant adipokine imbalance in metabolically healthy women is one of their metabolic health “protective” mechanisms.

Undoubtedly, genetic factors also play a role in the development of obesity in subjects with the different metabolic profile. There are evidences of the association between the metabolic syndrome and obesity with polymorphic variants of some genes, products of expression which play an important role in adipogenesis and the regulation of carbohydrate and lipid metabolism. Potential candidate genes include the adiponectin gene, leptin, and LEPR genes studied in our trial.

According to our study results, distribution of the G276G, G276T, and T276T genotypes and frequency of the 276T and 276G alleles of the leptin gene were not different in AO patients with or without metabolic disorders. In MUO patients and in MHO individuals, frequency of the 19G allele of this gene was 0.625 and 0.600, respectively, which corresponds to the incidence of this gene in the European population. Thus, frequency of this gene's 19G allele varies by different data from 0.36 to 0.67 being higher in Finns than in Frenchmen and Italians as far as the European population is concerned [16, 24, 25].

The paper by Hager et al. (1998) states that homozygotes of the 19A allele have lower leptin levels than carriers of the 19A allele [25]. In AO patients we evaluated, leptin levels were not different in various genotypes of the leptin gene. In 1999, Li et al. have found that A19G polymorphism of the leptin gene was a predictor of obesity (heterozygotes had higher BMI than homozygotes of the 19A and 19G alleles) although this association was not confirmed in other studies [26].

However, in a number of studies no differences were identified in leptin levels or associations with BMI, waist-to-hip ratio, body mass, and body fat in carriers of different genotypes of the leptin gene [16, 24, 27]. The results obtained in this study also have not found any correlation between obesity and metabolic disorders in different genotypes of the leptin genes.

At present, three single-nucleotide polymorphisms of the LEPR gene are known to lead to amino acid substitution in the receptor protein: Lys109Arg (K109R) rs1137100; Gln223Arg Rs1137101 (Q223R); and Lys656Asn (K656N) rs8179187 [28–30].

A668G polymorphism of the leptin receptor gene is studied most frequently. A668G polymorphism is localized in exon 6 of the extracellular region of the leptin receptor's C domain that has a leptin-binding area, which leads to a single amino acid substitution, that of glutamine (Gln) with arginine (Arg) at position 223, and accounts for the change in functional activity of the leptin receptor [31–33]. In the literature, this polymorphism is most often referred to as Q223R whereas the allele types of the leptin receptor gene are dubbed 223Q and 223R. Frequency of the 223Q and 223R alleles of this gene is highly variable across countries and ethnic groups. In particular, frequency of the 223R allele in Asian people is notably higher than that in other ethnic groups, up to 0.85 [33, 34]. Frequency of the 223R allele in healthy Europeans, by data of various authors, is in the range from 0.41 (England) to 0.44 (the Netherlands) [35–37]. For AO patients evaluated by us with and without metabolic disorders, frequency of the 223R allele of the LEPR gene was low and amounted to 0.438 and 0.410, respectively.

In our study we also found that in metabolically healthy and unhealthy women, carriers of the 223R allele of the leptin receptor gene, leptin level was higher than that in individuals with the Q223Q genotype. Furthermore, in MUO women with the R223R genotype, leptin level was higher than that in women who are carriers of the 223Q allele of the leptin receptor gene. In MHO women with different genotypes of the LEPR gene, leptin levels were not different (*p* > 0.05). These phenomena were not seen in men.

It has been noted in a number of studies that carriage of the 223R allele of the leptin receptor gene was associated with a high level of circulating leptin and reduced sensitivity of the leptin receptor [30, 32, 36] as well as increased BMI [36, 38, 39]. Other researchers received the data on the association of the R223 allele with glucose and insulin levels [40, 41]. Gottlieb et al. (2009) have found the correlation of this polymorphism with the metabolic syndrome: Q223Q and Q223R genotypes are more frequently encountered in MetS patients than in healthy individuals [42].

In the study carried out by us earlier, it was observed that in AO patients with MS/metabolic disorders carriership of the R223R genotype of the leptin receptor gene was associated with higher BMI and insulin levels; in men it was also associated with larger waist circumference [43]. In the study by van der Vleuten et al. (2006), it has been also shown that carriage of the 223R allele of the LEPR gene (homozygotes of the 223R alleles and heterozygotes) was associated with combined hyperlipidemia, reduced sensitivity to insulin and adiposity [44].

It is known that the adiponectin level is genetically controlled. Data of different authors suggest that heritability of the adiponectin level varies from 55% to 93% [45, 46].

Correlation between polymorphisms of the adiponectin gene and its production is identified, which may contribute to incidence of obesity, insulin resistance, type 2 diabetes mellitus, MS, and cardiovascular disease [47–51].

At present, single polymorphisms T45G and G276T are the most frequent ones among described polymorphisms of the adiponectin gene (Y111H, G-12823A, A-11426G, G-11391A, C11377G, A11426G, R112C, I164T, T45G, and G276T) [52]. Results of the studies devoted to these polymorphisms are controversial and largely dependent on studied cohorts.

In case of G276T (intron 2, rs1501299) polymorphism of the adiponectin gene, frequency of the 276G allele in the general population varies, by different data, from 0.701 to 0.830 [18, 48, 53–55]. According to our data, frequency of the 276G allele in AO patients with and without metabolic disorders did not differ (0.694 and 0.689). In a number of studies, association of the 276G allele with the low adiponectin level (France, Greece, Spain, and Japan), insulin resistance (Japan, Greece, Sweden, Spain, and Italy), obesity (Sweden, Japan), and T2DM (Japan) has been found [48, 53–57]. Yang et al. (2007) have found the association of the 276G allele with MS [55]. In our study, no differences in adiponectin levels in carriers of the 276G and 276T alleles have been observed.

With T45G (Gly45Cly) polymorphism (exon 2, rs2241766) of the adiponectin gene, frequency of the 45T allele in the general population, based on data of different authors, varies from 0.705 to 0.950 [18, 48, 53, 55, 58].

In a number of studies, relationship of T45G polymorphism and obesity, T2DM, and hypoadiponectinemia was noted. Thus, carriage of the 45T allele (wild type) was associated with low adiponectin level in healthy volunteers in the studies carried out in France and in Canada [56, 58]. Similar results were obtained in healthy individuals and patients with T2DM in Japan, Sweden, and Spain and Italy [48, 53, 57, 59]. Besides, in studies conducted in Sweden [57] and in Germany [60], carriage of the 45G allele was associated with adiposity. However, this association has not been found in studies carried out in Finland and in the multicenter trial done in Europe and Canada (STOP-NIDDM-study) [46, 61]. Carriage of the 45G allele has been also associated with increased risk of T2DM, impaired glucose tolerance, higher BMI and waist-to-hip ratio in the DESIR study (France) [62], increased T2DM risk (Japan) [48], and hyperinsulinemia and insulin resistance (Germany) [60].

Menzaghi et al. (2002) stated that the T45T genotype of the adiponectin gene was associated with insulin resistance and systolic blood pressure [53]. However, some researchers did not find any association between adiponectin gene polymorphism and insulin resistance and BMI [55, 63].

In the MetS patient population there were more carriers of the 45T allele of the adiponectin gene than among those metabolically healthy. Furthermore, T45T genotype of the adiponectin gene was associated with over twofold increased risk of metabolic disorders in AO patients even though adiponectin levels in AO patients with various genotypes of this gene were the same. Even when the groups were adjusted by excluding patients with class II and III obesity from MUO group the risk of metabolic disorders in AO patients had remained (OR = 2.496; 95% CI = 1.127 ÷ 5.524). Therefore, it can be assumed that carriers of T45T adiponectin gene genotype have a direct adverse effect on the metabolic profile in patients with AO. Also, as it was found in our previous trial, T45T genotype of the adiponectin gene and reduced adiponectin level in women were predictors of metabolic syndrome in AO patients [43]. Thus, lower frequency of the potentially unfavorable genotype of the adiponectin gene was found in metabolically healthy individuals, which, to some extent, might determine their metabolically favorable profile.

## 5. Conclusions

Prevalence of metabolically healthy obese individuals among patients with abdominal obesity is low, 8.7%. Benign metabolic status was associated with younger age, lower waist circumference, higher physical activity, and shorter duration of obesity. In women with uncomplicated abdominal obesity the leptin level was lower and the adiponectin level was higher than in women with complicated obesity. In metabolically healthy individuals with abdominal obesity low frequency of T45T adiponectin gene polymorphism was found, which is associated with an increased risk of metabolic syndrome in patients with abdominal obesity.

## Figures and Tables

**Table 1:** Anthropometric parameters and laboratory parameters in metabolically healthy and unhealthy patients with abdominal obesity.

Parameters	Healthy AO	Unhealthy AO	*p*
	(*n* = 44)	(*n* = 459)	
Age (yrs)	42.2 ± 1.4	47.0 ± 0.4	0.001
BMI, kg/m^2^	29.094 ± 0.511	31.561 ± 0.242	0.011
WC, сm			
M	104.65 ± 0.82	108.62 ± 0.95	0.013
F	93.29 ± 0.76	98.94 ± 0.65	0.014
WHR	0.857 ± 0.011	0.890 ± 0.004	0.002
SBP, mm Hg	113.28 ± 1.30	136.42 ± 0.88	0.0001
DBP, mm Hg	73.07 ± 1.08	86.75 ± 0.54	0.0001
Glucose, mmol/L	5.001 ± 0.072	5.624 ± 0.068	0.0001
Insulin, mIU/mL	15.189 ± 1.464	20.221 ± 0.776	0.024
HOMA-IR	3.464 ± 0.432	4.730 ± 0.245	0.043
TC, mmol/L	5.375 ± 0.123	5.866 ± 0.056	0.024
HDL-C, mmol/L	1.642 ± 0.061	1.212 ± 0.021	0.001
LDL-C, mmol/L	3.419 ± 0.130	3.955 ± 0.059	0.005
TG, mmol/L	1.025 ± 0.054	1.620 ± 0.042	0.0001
hsCRP, mg/L	3.009 ± 0.465	4.279 ± 0.180	0.012
Leptin, ng/mL			
M	29.304 ± 2.351	35.454 ± 3.410	NS
F	42.360 ± 3.101	57.504 ± 2.054	0.030
Adiponectin, mcg/mL			
M	17.330 ± 3.241	16.880 ± 0.851	NS
F	23.651 ± 0.864	19.014 ± 0.861	0.020

BMI, body mass index; WHR, waist-to-hip ratio; SBP, systolic blood pressure; DBP, diastolic blood pressure; HOMA-IR, homeostasis model assessment of insulin resistance; hsCRP, high-sensitivity C-reactive protein.

**Table 2:** Associations between waist circumference and metabolic parameters, blood pressure, and adipocytokines in patients with abdominal obesity (correlation analysis).

Indicators	*r*	*p*
Systolic BP	0.348	0.0001
Diastolic BP	0.231	0.0001
Glucose	0.244	0.0001
Insulin	0.405	0.0001
HOMA-IR	0.402	0.0001
HDL-C	−0.327	0.0001
TG	0.219	0.0001
Leptin	0.326	0.0001
Adiponectin	−0.214	0.003
hsCRP	0.223	0.0001

**Table 3:** Distribution of the G19G, G19A, and A19A genotypes and frequency of the 19A and 19G alleles of the leptin gene; distribution of the Q223Q, R223Q, and R223R genotypes and frequency of the 223Q and 223R alleles of the leptin receptor gene in patients with abdominal obesity.

Cohorts of AO patients	LEP genotypes			Allele frequency	
	G19G	G19A	A19A	19A	19G
MHO	13	22	5	0.400	0.600
MUO	169	186	64	0.375	0.625
*p*	NS	NS	NS	NS	NS

Cohorts of AO patients	LEPR genotypes			Allele frequency	
	Q223Q	R223Q	R223R	223Q	223R

MHO	14	18	7	0.590	0.410
MUO	108	245	57	0.562	0.438
*p*	NS	NS	NS	NS	NS

**Table 4:** Distribution of the G276G, G276T, and T276T genotypes and frequency of the 276T and 276G alleles; distribution of the G45G, G45T, and T45T genotypes and frequency of the 45G and 45T alleles of the adiponectin gene in patients with abdominal obesity.

Cohorts of AO patients	Adiponectin genotypes			Allele frequency	
	G276G	G276T	T276T	276T	276G
MHO	18	15	4	0.311	0.689
MUO	193	148	44	0.306	0.694
*p*	NS	NS	NS	NS	NS

Cohorts of AO patients	Adiponectin genotypes			Allele frequency	
	G45G	G45T	T45T	45T	45G

MHO	0	10	30	0.875	0.125
MUO	1	52	365	0.935	0.064
*p*	NS	0.034	0.042	0.005	0.005
